# Performance Characterization and Validation of Saliva as an Alternative Specimen Source for Detecting Hereditary Breast Cancer Mutations by Next Generation Sequencing

**DOI:** 10.1155/2016/2059041

**Published:** 2016-10-13

**Authors:** Varsha Meghnani, Nadeem Mohammed, Christopher Giauque, Rahul Nahire, Thomas David

**Affiliations:** Castle Medical, LLC, 5700 Highlands Parkway, Suite No. 100, Smyrna, GA 30082, USA

## Abstract

Identification of pathogenic germline mutations by next generation sequencing is a widely accepted tool for predicting the risk of hereditary cancer development. Blood is the most common source of DNA for such tests. However, blood as a sample type has many drawbacks, including the invasive collection method, poor sample stability, and a relatively high cost of collection. Therefore, in the current study we have assessed the suitability of saliva as an alternative source of genomic DNA for the identification of germline mutations in the BRCA1/2 genes by next generation sequencing (NGS). Our results show that all of the samples yielded DNA concentrations sufficient for library preparation. The concentrations of the final libraries, which were generated by PCR using target specific primers, fall into the expected range with no notable difference between libraries generated from DNA derived from saliva or blood. Quality parameters indicate that sequencing performance is comparable across sample source. An average of (98 ± 0.02)% variant calling concordance was obtained between the two specimen sources. Our data recommends saliva as a potential alternative for detecting germline mutation by next generation sequencing.

## 1. Introduction

Genetic tests for predicting the risk of hereditary breast and ovarian cancer have contributed towards reduction in cancer-related deaths. According to the National Cancer Institute, a significant drop in the deaths associated with breast and ovarian cancer has been observed since the year 2000 in the United States [1, 2]. The tumor suppressor genes* BRCA1* and* BRCA2* are the two major breast and ovarian cancer susceptibility genes, and deleterious mutations in these genes have shown to contribute in the pathogenesis of breast/ovarian cancer [3]. Approximately 20–25% of hereditary breast cancers are associated with mutations in* BRCA1* and* BRCA2* genes [4]. Genetic testing to confirm the presence of hereditary mutations in these genes can help patients take proper preventative measures, resulting in fewer cancer deaths.

Most of the commercial and noncommercial labs use whole blood as primary sample source for BRCA genetic tests. However, saliva has emerged as a more convenient alternative source of genomic DNA for genetic testing [5, 6]. The saliva collection process is simple and can generally be performed with minimal training or assistance. In contrast, collection of blood samples requires a specialized phlebotomist, while the associated pain, stress, and anxiety can make it more difficult to achieve patient compliance. This is particularly problematic in the case of risk-predicting genetic tests like BRCA testing, where the person being tested is often perfectly healthy. The necessity of getting blood drawn in such cases could deter some potential patients from consenting to undergo testing. Another major benefit associated with saliva as a genomic DNA source is its stability during storage and transport. Blood generally needs to be kept refrigerated before processing for DNA extraction, and storage and shipment conditions can adversely affect the yield and overall quality of the extracted DNA. Saliva collection kits, on the other hand, are available with stabilizing solutions that make them stable even at ambient temperature for a much longer period of time [7, 8]. Because of these advantages offered by saliva, scientists and clinicians are testing its suitability as a source for genomic DNA. Comparison studies performed on saliva and blood samples from matched donors have reported more than 97% concordance in SNP genotyping results [6, 9], but until now, to the best of our knowledge, there are no reports of performance comparison of the two sample types in next generation sequencing assays. In the present study, we compare the performance of saliva and blood as germline DNA sources for detecting hereditary BRCA mutations by Ion Torrent next generation sequencing.

## 2. Materials and Methods

### 2.1. Sample Collection

Six healthy volunteers of different age groups were selected from among the employees of Castle Medical, LLC. Informed consent was obtained from each volunteer before sample collection. Samples were deidentified by assigning them a unique code number before processing. Saliva and blood samples were collected from each volunteer as per the laboratory protocol under the supervision of certified clinician. Saliva samples were collected using the OraCollect® swabs (DNA Genotek, Ontario, Canada) as per the manufacturer instructions. Each volunteer was asked to hold the swab in the buccal cavity for at least 30 secs each side (total of 1 min) to soak up sufficient saliva. Saliva-soaked swabs were then mixed with 1 mL of bacteriostatic solution provided in the kit. Whole blood samples were collected in EDTA-containing lavender top tubes (Becton Dickinson, NJ).

### 2.2. DNA Extraction

Genomic DNA was extracted from both blood and saliva samples using Mag-Bind® paramagnetic particles (Mag-Bind Blood & Tissue DNA HDQ kit, Omega Biotek, GA) on a Biomek® NX^*P*^ liquid handler (Beckman Coulter). In brief, 400 *μ*L of each saliva sample (diluted with OraCollect bacteriostatic solution) or 200 *μ*L of each EDTA-containing whole blood sample was processed for the extraction according to the instructions provided in the kit. Extracted DNA samples from both specimen types were eluted in 50 *μ*L of elution buffer and were quantified by measuring their UV absorbance at wavelength 260 nm on a spectrophotometer (SpectraMax Plus). DNA samples were also quantified on a Qubit® 3.0 fluorometer (Thermo Fisher Scientific, CA) using the Qubit dsDNA HS quantification kit (Thermo Fisher), according to the manufacturer's instructions. The purity and quality of the DNA samples were assessed by reading each sample's OD at 260 nm and 280 nm on the spectrophotometer. The OD_260_/OD_280_ ratio for each DNA sample was calculated, and a ratio above 1.7 was considered sufficient purity [10]. After extraction, all the DNA samples were stored at −20°C.

### 2.3. Amplicon Library Construction

Target regions of BRCA1 and BRCA2 genes were amplified by a panel of specific primer pools designed using Ampliseq Designer™ software (Thermo Fisher Scientific, CA). The designed panel consists of 3 pools of primers. Each pool generates libraries of ~66 amplicons with sizes ranging between 125 and 275 bp. In brief, 30 ng of each DNA sample was amplified separately using 5x Ion Ampliseq High Fidelity master mix and Ampliseq primers, with thermocycling conditions determined by the manufacturer's protocol. This step was followed by partial digestion of primers from the amplified product using FuPa reagent (Thermo Fisher). For sample tracking and identification, each amplified library was tagged with a unique Ion Xpress Barcode (Thermo Fisher). After purification, amplicon libraries were quantified on a Qubit 3.0 fluorometer (Thermo Fisher) according to the manufacturer's instructions. Stock libraries were stored at −20°C until next step.

### 2.4. Enriched Templated Ion Sphere Particles Preparation

After normalization to 100 pM, all the libraries were pooled together in equal proportions and immediately processed for emulsion PCR on an Ion One Touch™ system to generate templated Ion sphere particles (ISPs) using an Ion One Touch 200 template kit v2 (Thermo Fisher). Templated ISPs were enriched using streptavidin MyOne beads on an Ion OneTouch ES system as per manufacturer instructions. Enriched templated ISPs were immediately processed for sequencing. Using the same set of libraries, we prepared fresh templated ISPs and sequenced all the samples twice on two different days.

### 2.5. Sequencing on Ion Torrent PGM System

Enriched templated ISPs were loaded on an Ion 314 Chip v2 (Thermo Fisher) after primer annealing and incubation with polymerase as per the instructions provided by the manufacture. The loaded chip was run on an Ion PGM Sequencer (Thermo Fisher) using the reagents provided in the Ion PGM Sequencing 200 kit (Thermo Fisher). Sequencing was performed with 500 flows which generated reads of about 200 bp length.

### 2.6. Data Analysis and Interpretation

Data generated from the PGM were analyzed and processed on Torrent Suite software version 5.0.2 (Thermo Fisher). After signal processing, base calling, and trimming of low quality reads, the data were aligned with the hg19 human reference genome (Genome reference Consortium GRCh37). Aligned files were then further analyzed for quality scoring, coverage analysis, and variant calling using Torrent Suite plugins. For the Variant Caller plugin, we used customized parameters optimized for Ion Ampliseq™ BRCA1 and BRCA2 Panel (Thermo Fisher). All the variants called by the Variant Caller plugin were quality checked using the Integrative Genomic Viewer to eliminate any artifacts and false positives due to poor quality data. Following raw data analysis, the variants were annotated by Ion Reporter™ software (Thermo Fisher). Variants were classified as “benign,” “unknown significance,” or “pathogenic” based on the information available at ClinVar, the BIC database, and the ARUP BRCA1/2 database.

Percentage concordance in the detected variants from blood-derived DNA libraries and their saliva-derived counterparts was calculated using the following equation [11]:

(1) Percentage concordance =2∗Number of overlapping variants(Sum of variants from both the samples).

### 2.7. Cross Validation of Data

To further confirm the accuracy of our data we collected an additional tube of blood from 5 out of the 6 volunteers in purple top EDTA-containing tubes. These blood samples were sent to a commercial reference lab for BRCA1/2 sequencing. The entire coding region of BRCA1/2 genes and flanking noncoding regions were analyzed by Miseq next generation sequencing platform using Truseq custom library prep reagents (Illumina). The variants reported by the reference lab for each sample were compared with the corresponding Variant Caller results generated in our lab. We compared variant type, zygosity, and clinical classification of each variant detected. Percentage concordance between the Variant Caller results generated using our sequencing platform and the corresponding results obtained from reference laboratory was calculated using the same equation as mentioned before.

## 3. Results

### 3.1. DNA Concentration and Quality

The concentrations of extracted DNA samples from blood samples were significantly higher than from paired saliva samples (*p* < 0.01), despite the extraction volume of the saliva samples being double that of the blood samples (200 versus 400 *μ*L). As shown in Table 1, we obtained an average of 91.7 ± 21 ng/*μ*L and 173.7 ± 64.09 ng/*μ*L DNA from blood samples when quantified by Qubit and spectrophotometer, respectively. The paired saliva samples gave an average of 15.83 ± 4.5 ng/*μ*L and 28.00 ± 9.3 ng/*μ*L of DNA samples upon quantification with Qubit and spectrophotometer, respectively. The quality of the extracted DNA, as determined by OD_260_/OD_280_ (≥1.7), were comparable from both specimen sources, suggesting limited amount of protein contamination in the DNA samples extracted from either of the sources.

### 3.2. Ampliseq Library Concentration

After normalization, all of the DNA samples were amplified using target specific primers to generate* BRCA1/2* amplified libraries. Each library was tagged with a unique barcode as explained in Materials and Methods. The amplified libraries as quantified by Qubit resulted in highly variable concentration from sample to sample (Table 1). There was little correlation observed between final library concentration and original sample source (*r* = 0.33), suggesting that factors other than input DNA source are responsible for the majority of the variability.

### 3.3. Data Quality after Alignment

The data generated by the sequencing run was evaluated by multiple quality parameters, including percentage of sequence aligned with the human genome, mean raw accuracy, and the quality of control test fragments. We found ≥98% of our data points aligned to the human genome with 99.3% mean raw accuracy. We also calculated the percentage of total bases called with ≥ Q20 (Phred quality score) for each sample and compared the calculated values for DNA samples obtained from both sources. Our calculated data indicates that DNA samples from both specimen types generated base calls with comparable accuracy. Mean depth of coverage and uniformity in depth coverage were quite variable irrespective of sample type. All samples were sequenced with at least 70x mean depth coverage and 91% coverage uniformity. No significant difference between blood and saliva samples was detected for any measure of data quality (Table 2).

### 3.4. Variant Caller Data from Matched Saliva, Blood and Reference Lab

Data generated from our Variant Caller plugin for saliva, blood, and data obtained from reference lab from matched donors were compiled as shown in Tables 3 [Table tab4]–5. Each table consists of ClinVar IDs (nucleotide change) of the variants detected and their zygosities for all three specimen types.

### 3.5. Concordance in Variant Caller Data

Variants detected in DNA extracted from saliva samples were 98% concordant with those from matched blood samples, while Variant Caller data generated by our sequencing platform was 89% concordant with the Variant Caller data obtained from the reference lab (Table 6). However, if we consider only exonic variants in the concordance calculation (omitted intronic, 5′-UTR, and 3′-UTR variants) we obtained an average of 100% concordance. These data suggest that the lower concordance value obtained in previous calculation is due to the fact that the reference lab did not report intronic and other UTR variants, which were detected by our platform.

## 4. Discussion

Next generation sequencing can be a time consuming, laborious and relatively expensive method, so it is crucial to do quality checks at each step to avoid needless repetitions. Failure to do so can negatively impact the quality of downstream data [12]. Since DNA extraction is the first step in the entire NGS workflow, it is important to test the quality and quantity of the extracted DNA before processing it further. Specimen source is one of the major factors that affects the quantity and quality of the isolated DNA. In our study, saliva-derived DNA samples yielded at significantly lower DNA concentrations than their paired blood-derived DNA samples, by both Qubit and spectrophotometer (*p* < 0.01). Dilution of saliva in the bacteriostatic solution provided in the kit is one cause of the lower yield of DNA from saliva samples, but another possible explanation is the presence of fewer DNA-containing cells in saliva than in a comparable volume of blood. Such a reduction in yield from saliva compared to blood has been previously reported elsewhere [6].

The DNA concentrations measured by the spectrophotometer were noticeably higher than those given by the Qubit fluorometer for all specimens. Contamination of genomic DNA with fragmented DNA or RNA can lead to overestimation in quantification by spectrophotometer [12, 13]. Therefore, we used the Qubit readings, which should directly reflect the concentration of double-stranded DNA, as our final estimate of DNA concentration in all samples. Our OD260/OD280 ratio data show a variable amount of protein contamination in all of the extracted DNA samples irrespective of the sample source. The purity and quality of the source DNA can adversely affect the NGS data quality. While the lower concentration of DNA obtained from saliva samples may pose a challenge for some methods, such as the detection of low-frequency somatic mutations [14], the yield from saliva proved to be sufficient for the detection of germline BRCA variants by NGS in this study. It should be noted that apart from the specimen source, other external factors such as the collection device, specimen quality, transportation conditions, and extraction reagents can also influence DNA yield [15]. The yield of DNA from saliva can be improved by adopting better collection and extraction parameters. For example, in this study, OraCollect devices were used for saliva collection due to their lower cost, but other collection devices like OraGene (Genotek) have been proven to result in superior DNA yield.

Previously published reports have noted that saliva samples, unlike blood, can include bacterial cells, and the presence of microbial DNA in the sample can lead to an overestimation of the amount of extracted patient DNA [6, 16]. In this study, we have not confirmed nor quantified any bacterial DNA in our samples. Since the extracted DNA samples serve as templates for human-specific primers in generating the final BRCA1/2 libraries, an incorrect estimate of initial DNA concentration could directly affect the final concentration of the generated libraries. However, in our study no significant difference was observed between the concentrations of libraries generated by DNA samples derived from saliva and from their paired blood samples. These data suggest that sufficient human DNA was present in the saliva samples to serve as PCR template. We suspect that the activity of the bacteriostatic solution in the OraCollect kit may have helped to minimize the microbial load of saliva samples.

In the present study, no correlation was observed between the specimen source and the quality score, mean depth coverage, or the sequence uniformity values, although all of these quality control parameters were highly variable from sample to sample. Despite this variability, the quality control results for all samples irrespective of their specimen source satisfied our previously established minimum acceptability criteria (mean coverage ≥ 50x; coverage uniformity ≥ 90%; percent data alignment ≥ 98%; mean raw accuracy ≥ 90%). Our quality matrix data suggest that DNA extracted from saliva samples is as suitable for the generation of high quality NGS data as is DNA derived from whole blood.

Variant Caller data generated from saliva samples were highly concordant with the blood samples of matched donors, again supporting the use of saliva as a suitable source of genomic DNA. Buccal epithelial cells and leukocytes are the two major cellular components of saliva [17]. Previous studies have reported that leukocytes are the major source of genomic DNA in saliva, which is also true for blood; thus suggesting that saliva is comparable to blood as a source of genomic DNA for many applications [18]. Based on our inspection of the data, we believe that the few nonconcordant variants were most likely artifacts that arose during the library preparation and sequencing process. It is known that in addition to DNA quantity and integrity, NGS data can also be affected by factors such as platform specific error, PCR amplification bias, and sequencing bias.

Our Variant Caller data generated from blood samples were further cross validated by an independent reference lab. We obtained less than 90% concordance in the data generated in-house and that generated by the reference lab for the matched donors which was improved to 100% if only exonic variants were considered in the calculation. Although, the method used by the reference lab claims to detect all the noncoding variants, however they have not reported some of the intronic variants. This implies that lesser number of variants have been detected by Miseq system as compared to the Ion Torrent system, which is in agreement with the previously published study [19].

In summary, we have compared the next generation sequencing results obtained from DNA derived from saliva to those derived from whole blood samples. With this study we conclude that saliva can be used as an alternative to blood for detecting germline mutations by NGS methods without compromising data quality. The lower DNA yield obtained from saliva may be one reason why it is not currently as widely accepted as blood for NGS-based testing, but our data strongly suggest that at least for some applications, the yield is sufficient to make saliva an acceptable and attractive alternative. Further work is required to more clearly establish the effect of different collection and extraction methods on the final data quality. In addition, a larger sample size comprising a wider range of variants, including pathological variants and indels, is needed to verify that all clinically relevant genetic variants can be detected as reliably in saliva samples as they are in blood samples. Finally, the suitability of saliva as a source of DNA for other NGS-based testing would need to be individually validated on a test-by-test basis.

## Figures and Tables

**Table 1:** Concentration of DNA samples extracted from paired blood and saliva as quantified by Qubit and spectrophotometer. Sample tracking Ion Xpress barcodes and concentrations of Ampliseq libraries generated by each DNA sample.

Sample ID	DNA Qubit quantification		DNA spectrophotometer quantification		OD260/OD280	Ion Xpress barcode (s)	Library concentration (ng/mL)
	Concentration (ng/*µ*L)	Total yield (*µ*g)	Concentration (ng/*µ*L)	Total yield (*µ*g)			
1601074001Saliva	20.0	1.0	38.4	1.9	1.808	13	148
1601074001Blood	97.4	4.8	148.5	7.4	1.724	14	191
1601074002Saliva	18.3	0.9	29.4	1.4	1.942	11	528
1601074002Blood	110.0	5.5	166.6	8.3	1.744	12	139
1601074003Saliva	10.9	0.5	27.2	1.3	1.865	11	290
1601074003Blood	57.4	2.8	291.6	14.5	1.913	10	476
1601074004Saliva	10.8	0.5	15.8	0.7	1.945	13	185
1601074004 Blood	93.2	4.6	105.6	5.2	1.847	12	75.4
1601074005Saliva	14.2	0.7	19.0	0.9	1.895	9	77.4
1601074005 Blood	78.2	3.9	189.2	9.4	1.891	8	69.2
1601074006 Saliva	20.8	1.0	37.7	1.8	1.843	7	50
1601074006Blood	114.0	5.7	140.7	7	1.88	6	58.8
*Mean (blood)*	*91.7*	*4.6*	*173.7*	*8.6*	*1.88*		*168.2*
*Mean (saliva)*	*15.8*	*0.8*	*27.9*	*1.3*	*1.83*		*213*
*p value: blood versus saliva (T test)*	>0.001		>0.01				

**Table 2:** Quality score, mean depth coverage, and uniformity of the data generated from DNA samples extracted from saliva and their matched blood samples.

Sample ID	Percentage of data ≥ Q20	Mean depth of coverage	Uniformity
1601074001Saliva	91.32%	246.8	92.96%
1601074001Blood	91.35%	246.3	98.59%
1601074002Saliva	91.05%	785.8	97.60%
1601074002Blood	91.32%	442.1	97.41%
1601074003Saliva	92.99%	218.2	96.71%
1601074003Blood	92.78%	82.2	98.20%
1601074004Saliva	92.68%	375.2	97.07%
1601074004Blood	93.00%	76.8	94.39%
1601074005Saliva	92.93%	139.9	93.02%
1601074005Blood	92.62%	212.2	96.63%
1601074006Saliva	92.83%	84.5	90.96%
1601074006Blood	92.61%	123.8	98.33%
*Mean blood*	*92.28%*	*197.2*	*97.26%*
*Mean saliva*	*92.30%*	*308.4*	*94.72%*

**Table 3:** Variant Caller data from saliva and from corresponding blood sample for sample IDs 1601074001 and 1601074002.

1601074001Saliva		1601074001Blood		1601074002Saliva		1601074002Blood		1601074002Blood reference lab	
Nucleotide	Zygosity	Nucleotide	Zygosity	Nucleotide	Zygosity	Nucleotide	Zygosity	Nucleotide	Zygosity
c.1114A>C	Hetero	c.1114A>C	Hetero	c.-26G>A	Hetero	c.-26G>A	Hetero	—	—
c.3807T>C	Hetero	c.3807T>C	Hetero	c.1114A>C	Hetero	c.1114A>C	Hetero	c.1114A>C	Hetero
c.4563A>G	Homo	c.4563A>G	Homo	c.3396A>G	Hetero	c.3396A>G	Hetero	c.3396A>G	Hetero
c.6513G>C	Homo	c.6513G>C	Homo	c.4563A>G	Homo	c.4563A>G	Homo	c.4563A>G	Homo
c.7397C>T	Homo	c.7397C>T	Homo	c.6153G>C	Homo	c.6153G>C	Homo	c.6153G>C	Homo
c.7469T>C	Hetero	c.7469T>C	Hetero	c.7397C>T	Homo	c.7397C>T	Homo	c.7397C>T	Homo
c.1067A>G	Hetero	c.1067A>G	Hetero	c.4837A>G	Hetero	c.4837A>G	Hetero	c.4837A>G	Hetero
c.442-34C>T	Homo	c.442-34C>T	Homo	c.4308T>C	Hetero	c.4308T>C	Hetero	c.4308T>C	Hetero
				c.3548A>G	Hetero	c.3548A>G	Hetero	c.3548A>G	Hetero
				c.3113A>G	Hetero	c.3113A>G	Hetero	c.3113A>G	Hetero
				c.2612C>T	Hetero	c.2612C>T	Hetero	c.2612C>T	Hetero
				c.2311T>C	Hetero	c.2311T>C	Hetero	c.2311T>C	Hetero
				c.2082C>T	Hetero	c.2082C>T	Hetero	c.2082C>T	Hetero

**Table 4 tab4:** Variant Caller data from saliva and from corresponding blood sample for sample ID 1601074003 and 1601074004.

1601074003Saliva		1601074003Blood		1601074003Blood reference lab		1601074004Saliva		1601074004Blood		1601074004Blood reference lab	
Nucleotide	Zygosity	Nucleotide	Zygosity	Nucleotide	Zygosity	Nucleotide	Zygosity	Nucleotide	Zygosity	Nucleotide	Zygosity
c.-26G>A	Hetero	c.-26G>A	Hetero	—	—	c.1114A>C	Hetero	c.1114A>C	Hetero	c.1114A>C	Hetero
c.3396A>G	Hetero	c.3396A>G	Hetero	c.3396A>G	Hetero	c.3396A>G	Hetero	c.3396A>G	Hetero	c.3396A>G	Hetero
c.3807T>C	Hetero	c.3807T>C	Hetero	c.3807T>C	Hetero	c.4563A>G	Homo	c.4563A>G	Homo	c.4563A>G	Homo
c.4563A>G	Homo	c.4563A>G	Homo	c.4563A>G	Homo	c.6513G>C	Homo	c.6513G>C	Homo	c.6513G>C	Homo
c.6513G>C	Homo	c.6513G>C	Homo	c.6513G>C	Homo	c.7242A>G	Hetero	c.7242A>G	Hetero	c.7242A>G	Hetero
c.7242A>G	Hetero	c.7242A>G	Hetero	c.7242A>G	Hetero	c.7397C>T	Homo	c.7397C>T	Homo	c.7397C>T	Homo
c.7397C>T	Homo	c.7397C>T	Homo	c.7397C>T	Homo	c.7806-14T>C	Hetero	c.7806-14T>C	Hetero	c.7806-14T>C	Hetero
c.7806-14T>C	Hetero	c.7806-14T>C	Hetero	c.7806-14T>C	Hetero	c.8755-66T>C	Hetero	c.8755-66T>C	Hetero	—	—
c.8755-66T>C	Hetero	c.8755-66T>C	Hetero	—	—	c.8830A>T	Hetero	c.8830A>T	Hetero	c.8830A>T	Hetero
c.9257-83G>A	Hetero	c.9257-83G>A	Hetero	—	—	c.^*∗*^36C>G	Hetero	c.^*∗*^36C>G	Hetero	—	—
c.10234A>G	Hetero	c.10234A>G	Hetero	c.10234A>G	Hetero	c.4837A>G	Hetero	c.4837A>G	Hetero	c.4837A>G	Hetero
None detected		None detected		None detected		c.3548A>G	Hetero	c.3548A>G	Hetero	c.3548A>G	Hetero
						c.2612C>T	Hetero	c.2612C>T	Hetero	c.2612C>T	Hetero
						c.2458A>G	Hetero	c.2458A>G	Hetero	c.2458A>G	Hetero
						c.2082C>T	Hetero	c.2082C>T	Hetero	c.2082C>T	Hetero
						c.212+23T>A	Hetero	c.212+23T>A	Hetero	—	—
						c.-19-115T>C	Hetero	—	—	—	—

**Table 5:** Variant Caller data from saliva and from corresponding blood sample for sample IDs 1601074005 and 1601074006.

1601074005Saliva		1601074005Blood		1601074005Blood reference lab		1601074006Saliva		1601074006Blood		1601074006Blood reference lab	
Nucleotide	Zygosity	Nucleotide	Zygosity	Nucleotide	Zygosity	Nucleotide	Zygosity	Nucleotide	Zygosity	Nucleotide	Zygosity
c.-26G>A	Hetero	c.-26G>A	Hetero	—	—	—	Hetero	c.-26G>A	Hetero	—	—
c.3396A>G	Hetero	c.3396A>G	Hetero	c.3396A>G	Hetero	c.681+56C>T	Hetero	c.681+56C>T	Hetero	—	—
c.4563A>G	Homo	c.4563A>G	Homo	c.4563A>G	Homo	c.1910-74T>C	Hetero	c.1910-74T>C	Hetero	—	—
c.6513G>C	Homo	c.6513G>C	Homo	c.6513G>C	Homo	c.3396A>G	Hetero	c.3396A>G	Hetero	c.3396A>G	Hetero
c.7242A>G	Hetero	c.7242A>G	Hetero	c.7242A>G	Hetero	c.4563A>G	Homo	c.4563A>G	Homo	c.4563A>G	Homo
c.7397C>T	Homo	c.7397C>T	Homo	c.7397C>T	Homo	c.6513G>C	Homo	c.6513G>C	Homo	c.6513G>C	Homo
c.7806-14T>C	Hetero	c.7806-14T>C	Hetero	c.7806-14T>C	Hetero	c.7397C>T	Homo	c.7397C>T	Homo	c.7397C>T	Homo
c.8487+19A>G	Hetero	c.8487+19A>G	Hetero	c.8487+19A>G	Hetero	c.7806-14T>C	Hetero	c.7806-14T>C	Hetero	c.7806-14T>C	Hetero
c.8755-66T>C	Hetero	c.8755-66T>C	Hetero	—	—	c.8755-66T>C	Hetero	c.8755-66T>C	Hetero	—	—
c.^*∗*^36C>G	Hetero	c.^*∗*^36C>G	Hetero	—	—	c.4837A>G	Hetero	c.4837A>G	Hetero	c.4837A>G	Hetero
c.4837A>G	Homo	c.4837A>G	Homo	c.4837A>G	Homo	c.4308T>C	Hetero	c.4308T>C	Hetero	c.4308T>C	Hetero
c.4308T>C	Hetero	c.4308T>C	Hetero	c.4308T>C	Hetero	c.3548A>G	Hetero	c.3548A>G	Hetero	c.3548A>G	Hetero
c.3548A>G	Homo	c.3548A>G	Homo	c.3548A>G	Homo	c.3113A>G	Hetero	c.3113A>G	Hetero	c.3113A>G	Hetero
c.3113A>G	Hetero	c.3113A>G	Hetero	c.3113A>G	Hetero	—	—	c.2612C>T	Hetero	c.2612C>T	Hetero
c.2612C>T	Homo	c.2612C>T	Homo	c.2612C>T	Homo	c.2311T>C	Hetero	c.2311T>C	Hetero	c.2311T>C	Hetero
c.2458A>G	Hetero	c.2458A>G	Hetero	c.2458A>G	Hetero	c.2082C>T	Hetero	c.2082C>T	Hetero	c.2082C>T	Hetero
c.2311T>C	Hetero	c.2311T>C	Hetero	c.2311T>C	Hetero	c.81-12delC	Homo	—	—		
c.2082C>T	Homo	c.2082C>T	Homo	c.2082C>T	Homo						
c.441+17T>C	Hetero	c.441+17T>C	Hetero	c.441+17T>C	Hetero						
c.212+23T>A	Hetero	c.212+23T>A	Hetero	—	—						

**Table 6:** Percentage concordance in the Variant Caller data from blood versus paired saliva and blood in-house data versus blood reference lab data.

Samples	Variant Caller data concordance In-house blood versus saliva	Variant Caller data concordance In-house blood versus reference lab blood	Variant Caller data concordance In-house blood versus reference lab blood (considering only exonic variants)
1601074001	100%	Data not available	Data not available
1601074002	100%	96%	100%
1601074003	100%	84.2%	100%
1601074004	96.9%	89.6%	100%
1601074005	100%	88.9%	100%
1601074006	90.3%	85.7%	100%
*Mean*	*97.9*	*88.9*	100%
